# A microRNA Signature Associated with Early Recurrence in Breast Cancer

**DOI:** 10.1371/journal.pone.0091884

**Published:** 2014-03-14

**Authors:** Luis G. Pérez-Rivas, José M. Jerez, Rosario Carmona, Vanessa de Luque, Luis Vicioso, M. Gonzalo Claros, Enrique Viguera, Bella Pajares, Alfonso Sánchez, Nuria Ribelles, Emilio Alba, José Lozano

**Affiliations:** 1 Laboratorio de Oncología Molecular, Servicio de Oncología Médica, Instituto de Biomedicina de Málaga (IBIMA), Hospital Universitario Virgen de la Victoria, Málaga, Spain; 2 Departamento de Lenguajes y Ciencias de la Computación, Universidad de Málaga, Málaga, Spain; 3 Plataforma Andaluza de Bioinformática, Universidad de Málaga, Málaga, Spain; 4 Servicio de Anatomía Patológica, Instituto de Biomedicina de Málaga (IBIMA), Hospital Universitario Virgen de la Victoria, Málaga, Spain; 5 Departmento de Biología Molecular y Bioquímica, Universidad de Málaga, Málaga, Spain; 6 Departmento of Biología Celular, Genética y Fisiología Animal, Universidad de Málaga, Málaga, Spain; University of Dundee, United Kingdom

## Abstract

Recurrent breast cancer occurring after the initial treatment is associated with poor outcome. A bimodal relapse pattern after surgery for primary tumor has been described with peaks of early and late recurrence occurring at about 2 and 5 years, respectively. Although several clinical and pathological features have been used to discriminate between low- and high-risk patients, the identification of molecular biomarkers with prognostic value remains an unmet need in the current management of breast cancer. Using microarray-based technology, we have performed a microRNA expression analysis in 71 primary breast tumors from patients that either remained disease-free at 5 years post-surgery (group A) or developed early (group B) or late (group C) recurrence. Unsupervised hierarchical clustering of microRNA expression data segregated tumors in two groups, mainly corresponding to patients with early recurrence and those with no recurrence. Microarray data analysis and RT-qPCR validation led to the identification of a set of 5 microRNAs (the 5-miRNA signature) differentially expressed between these two groups: miR-149, miR-10a, miR-20b, miR-30a-3p and miR-342-5p. All five microRNAs were down-regulated in tumors from patients with early recurrence. We show here that the 5-miRNA signature defines a high-risk group of patients with shorter relapse-free survival and has predictive value to discriminate non-relapsing versus early-relapsing patients (AUC = 0.993, p-value<0.05). Network analysis based on miRNA-target interactions curated by public databases suggests that down-regulation of the 5-miRNA signature in the subset of early-relapsing tumors would result in an overall increased proliferative and angiogenic capacity. In summary, we have identified a set of recurrence-related microRNAs with potential prognostic value to identify patients who will likely develop metastasis early after primary breast surgery.

## Introduction

Breast cancer comprises a group of heterogeneous diseases that can be classified based on both clinical and molecular features [1]–[5]. Improvements in the early detection of primary tumors and the development of novel targeted therapies, together with the systematic use of adjuvant chemotherapy, has drastically reduced mortality rates and increased disease-free survival (DFS) in breast cancer. Still, about one third of patients undergoing breast tumor excision will develop metastases, the major life-threatening event which is strongly associated with poor outcome [6], [7].

The risk of relapse after tumor resection is not constant over time. A detailed examination of large series of long-term follow-up studies over the last two decades reveals a bimodal hazard function with two peaks of early and late recurrence occurring at 1.5 and 5 years, respectively, followed by a nearly flat plateau in which the risk of relapse tends to zero [8]–[10]. A causal link between tumor surgery and the bimodal pattern of recurrence has been proposed by some investigators (i.e. an iatrogenic effect) [11]. According to that model, surgical removal of the primary breast tumor would accelerate the growth of dormant metastatic foci by altering the balance between circulating pro- and anti-angiogenic factors [9], [11]–[14]. Such hypothesis is supported by the fact that the two peaks of relapse are observed regardless other factors than surgery, such as the axillary nodal status, the type of surgery or the administration of adjuvant therapy. Although estrogen receptor (ER)-negative tumors are commonly associated with a higher risk of early relapse [15], the bimodal distribution pattern is observed with independence of the hormone receptor status [16]. Other studies also suggest that the dynamics of tumor relapse may be a consequence of the surgical procedure to remove the primary tumor, which would alter the circulating levels of VEGF, TNFα and several other inflammatory cytokines [17]–[19]. However, empirical evidence demonstrating a molecular link between surgery of the primary breast tumor and a bimodal pattern of recurrence is still lacking.

The identification of distinctive molecular portraits by microarray-based gene expression profiling has led to a breast tumor classification into five different subtypes: luminal A, luminal B, HER2 overexpressing (HER2+), basal-like and normal-like [3], [4]. Such classification has been adopted in the clinical routine, defining intrinsic subtypes with distinct histological characteristics, response to drug treatment and clinical outcome [3], [20]–[23]. HER2 and basal-like subtypes commonly associate with higher risk of relapse while luminal tumors frequently correlate with long-term DFS [24]–[26]. A recent study from the Molecular Taxonomy of Breast Cancer International Consortium (METABRIC) has proposed a new genome-driven classification of breast cancer by integrating both genomic and transcriptomic data. This new molecular stratification relies on the impact of somatic copy number aberrations (CNAs) on the transcriptome and classifies breast tumors in 10 integrative clusters (IntClusts 1–10), each associated with distinct clinical outcomes [27], [28].

MicroRNAs (miRNAs) are small, single-stranded RNAs with an important role in the regulation of gene expression [29], [30]. They are transcribed as large RNA precursors (pri-miRNAs) that are sequentially processed in the nucleus to produce a RNA hairpin of 65 nucleotides (nt), termed the precursor-miRNA (pre-miRNA) and in the cytoplasm to produce a 19–23 nt mature, active miRNA [31]–[33]. In general, miRNAs act as negative modulators of gene expression, binding to a partially complementary sequence usually located in the 3′-UTR region of their target mRNA and inhibiting its translation [34]. Due to this partial complementation, a single miRNA can target multiple transcripts, hence down-regulating many proteins in the same or in different pathways [29]. Currently, 1872 precursors and 2578 mature miRNAs have been identified in the human genome (miRBase 20, www.mirbase.org) [35], although the biological role of most of them remains to be determined.

As happens to mRNA expression, the transcriptional profile of miRNAs can vary among different tissues and stages of development. Alterations of miRNA patterns and sequences are common in several diseases, including cancer [36], [37]. They are involved in many deregulated pathways in tumor cells, especially those regarding the hallmarks of cancer [38], [39] and are often located in breakpoint regions that are amplified, deleted or translocated in cancer [36]. Several miRNAs have oncogenic (oncomiRs) or tumor suppressor (TS-miRs) activities and therefore, can contribute to tumorigenesis, tumor progression and metastasis [40]–[42].

In addition, miRNA expression profiles can provide molecular information of clinical relevance in cancer [43]. Thus, tumors of different origins can be classified according to particular sets of expressed miRNAs (signatures). Different subtypes of the same tumor can also be discriminated by miRNA expression and, in some cases, they allow predictive clinical evaluation [37], [44]–[46]. MicroRNAs are generally well-preserved in a wide range of specimen types, including body fluids and formalin-fixed paraffin-embedded (FFPE) tissues [47]. These two aspects have led to an active search for miRNAs with diagnostic and/or prognostic value. Several miRNAs have been linked to breast cancer metastasis [48], [49]. To our knowledge, however, the early peak of relapse, as defined by Demicheli et al. [9] has not been investigated as a separate, distinct entity from a molecular perspective. Thus, the following study was aimed at finding differences in miRNA expression patterns on a set of breast tumors from patients who developed early recurrence (≤24 months post-surgery), late recurrence (50–60 months post-surgery) and those who were free of disease after surgery during a 60-months follow-up. By performing a microarray-based study, we have identified a set of five miRNAs down-regulated in tumors from early-relapsing patients. We show here that the so-called 5-miRNA signature is associated with a shorter relapse-free survival (RFS) and has predictive value as determined by a ROC curve analysis. Although our study neither proves nor rules out a iatrogenic effect derived from surgery, it shows that non-relapsing and early-relapsing breast tumors can be distinguished at the molecular level by a disctint set of 5 miRNAs which likely determines their proliferative potential. Thus, the computational analysis of putative, experimentaly verified mRNA targets for the 5-miRNA signature and their associated gene ontology (GO) terms suggest that, at least in part, early recurrence in breast cancer is a consequence of an increased proliferative and angiogenic capacity of the primary tumor.

## Materials and Methods

### Patient material

The 75 patients included in this study underwent primary breast cancer surgery at the Hospital Universitario Virgen de la Victoria (HUVV, Málaga, Spain), between years 1998 and 2005. All patients provided written informed consent for inclusion in the study, which was approved by the hospital's Institutional Review Board (IRB). Patients were uniformly treated and followed according to the protocols established by the Clinical Oncology Department, based on scientific evidence and international recommendations. All clinical investigation were conducted according to the principles expressed in the Declaration of Helsinki.None of the patients received neoadjuvant therapy. Clinicopathological and follow-up information were obtained for each patient by chart review.

### Immunohistochemistry

Archived formalin-fixed and paraffin-embedded (FFPE) tumors (n = 75) were retrieved, a pathologist selected the most representative areas and tissue microarrays were constructed with triplicate cores (0.6 mm in diameter). Tumors were classified according to the intrinsic subtypes by immunohistochemical staining. Specific antibodies against estrogen receptor (ER, clone SP1), progesterone receptor (PR, clone Y85), Ki-67 (clone SP6) epidermal growth factor receptor 1 (EGFR1, clone EP38Y), vascular endothelial growth factor (VEGF, clone EP1176Y) and cytokeratin 5/6 (CK5/6, clone D5/16B4) were all purchased from Master Diagnostica (Spain). HER2 immunostaining was performed with the HercepTest™ (Dako, Denmark). Interpretation of immunohistochemical data was performed as previously described [23], [50], [51] by two pathologists blinded to the clinicopathological characteristics and the outcome of each patient.

### RNA extraction and microarray hybridization

FFPE tumor areas enriched with >90% breast cancer cells were selected and cells were manually microdissected from 3x10 μm sections. Total RNA was extracted using the RecoverAll Total Nucleic Acid Isolation kit (Life Technologies, Grand Island, NY, USA). RNA was converted to cDNA and hybridized to Affymetrix miRNA Chip array 2.0 (Affymetrix, Santa Clara, CA, USA). Chip hybridization and scanning were performed at the Functional Genomics Core facility (Institute for Research in Biomedicine, IRB Barcelona, Spain) following the recommendations of the manufacturer.

### Microarray data analysis

All statistical analyses were performed using the open-source R programming environment (v2.14.2) together with the open-source Bioconductor v2.10 libraries [52]. MicroRNA microarrays were first analyzed for quality control and expression of each miRNA feature was normalized and summarized across the remaining slides using the best-suited algorithm RMA [53]–[55]. Although the miRNA Chip Array 2.0 contanins probes representing 131 different organisms, only human features were used for further analyses. To increase the statistical power of the analyses, those miRNAs whose expression variability was below a threshold of 66% standard deviation were removed. Differential expression of normalized data was assessed using two R packages: *limma*, a moderated t-statistic based on an empirical Bayes approach [56] and *RankProd*, a simple non-parametric statistical method based on ranks of fold changes [57]. The multi-testing effect was corrected adjusting p-values by the Benjamini and Hochberg method. A gene was considered significantly up- or down-regulated when it complied the following two criteria: i) adjusted p≤0.05 and ii) fold change ≥2. The best candidates were those that appeared at both parametric and non-parametric tests. Independent comparisons were carried out for B *vs* A (B/A), BC *vs* A (BC/A), and B *vs* AC (B/AC). Group C alone could not be compared with A or B since microarray data from group C did not provide statistically significant differentially expressed miRNAs. The MIAME compliant microarray data has been deposited in the ArrayExpress public repository (EBI, UK), with accession number E-MTAB-1989.

### RT-qPCR validation

Ten nanograms of total RNA from each tumor sample were used to obtain cDNA by reverse transcription with specific miRNA primers and reagents from the TaqMan MicroRNA Reverse Transcription Kit (Life Technologies, Grand Island, NY, USA). PCR products were amplified from cDNA samples with the TaqMan MicroRNA Assays using the TaqMan Universal PCR Master Mix. All the assays were performed in triplicate according to the manufacturer's instructions. Relative miRNA expression was calculated using the ΔΔC_t_ method. The small RNAs RNU6b and miR-16 were used as reference for normalization.

### Survival analysis

The *survival* package in R was used to compute survival estimates and perform multivariate regression analysis [58]. Clinico-pathological and microRNA expression variables were analyzed and cumulative relapse-free survival (RFS), defined as time from surgery until recurrence, was considered as the clinical endpoint in survival outcomes. Patients without relapse or lost to follow-up were censored at last follow-up. Actuarial survival was performed by the Kaplan-Meier method and the significance in statistical differences was assessed using a class of rank test procedures for censored survival data (log-rank, Tarone-Ware and Peto-Peto tests). A Cox proportional hazard regression model [59] was used to examine the relationships of RFS and the prognostic factors, and all possible combinations of covariates were tested to identify the best model according to the Akaike Information Criterium (AIC) value [60], a measure of the relative quality of a statistical model. The assumption of hazard proportionality for the Cox models was checked by testing for non-slope in a generalized linear regression of the scaled Schoenfeld residuals on functions of time [60]. A non-zero slope indicated a violation of the proportional hazard assumption and the model was then excluded from the analysis.

### miRNAs signature prediction model

Three are the steps involved in the estimation of expression profiles to predict the outcome of future observations: model selection, prediction assessment and feature selection [61]. The naive Bayes classifier was used in this work to predict the class in future observations. The naive Bayes classifier is a standard model based on Bayes theorem with no domain-specific assumptions. With this model, a new sample would be classified into the most probable class based on posterior probability and computed according to the Bayes theorem. This classifier will be used to estimate predictive models in between-groups comparisons. The C-index is the most widely accepted measure of discrimination ability for a predictive model. In binary cases, this metric is equivalent to the area under the Receiver Operating Characteristic curve (AUC), which is commonly used to measure the predictive ability of logistic regression models.

A generalization of the 632+ bootstrap estimation of the misclassification error to estimate the TPR, FPR and ROC curves [62] was used to assess the prediction accuracy for the naive Bayes classifiers. The bootstrap estimator is obtained by drawing B bootstrap samples of size N with replacement. The observations in the bootstrap samples are used for training, while the remaining observations (the out-of-bag sample) are used for testing. Performance estimations are averaged for each prediction over all out-of-bag samples, and the bootstrap estimate of the TPR, FPR and ROC curve is defined analogously to the bootstrap error. The e1071 [52] and Daim (http://CRAN.R-project.org/package=Daim) packages were used in R to conduct these analyses. Feature selection refers to decide which miRNAs to include in the prediction, and it is a crucial step in developing a class predictor. Including too many features could reduce the model accuracy and may lead to a data overfitting [63]. To avoid it, all combinations for miRNAs were tested to identify the model containing the miRNA expression signature that more accurately predicted between groups at risk.

### miRNA target prediction

Validated targets for each miRNA were obtained from mirTarBase (http://mirtarbase.mbc.nctu.edu.tw/) [64] and miRecords (http://mirecords.biolead.org/) [65]. Both databases contain experimentally validated miRNA-target interactions (MTIs) curated by data mining of the published literature. The mirTarBase v4.3, includes a total 20,907 validated human MTIs for 384 miRNAs and 9,816 mRNAs. The CyTargetLinker plugin in Cytoscape [66] was used to retrieve validated and predicted MTIs for the five miRNAs identified in our study and to visualize them in a graphical way. The ClueGo and CluePedia plugins [67], [68] were used to retrieve the Gene Ontology (GO) annotations for the target genes identified with Cytargetlinker [69]. The enrichment of GO terms in the target genes set was assessed with a right-sided hypergeometric statistical analysis which provides a p-value that was further corrected using a Benferroni step down method. Only GO terms with corrected p-value≤0.01 were considered.

## Results and Discussion

### Microarray analysis

To identify miRNAs associated with early and late recurrence, the abundance of 1105 miRNAs was analyzed in a cohort of 75 primary breast tumors by the microarray technology. Tumors were classified into three prognosis groups according to the clinical outcome of the patients as follows: group A, patients who were disease-free ≥60 months after tumor excision; group B, patients who developed early recurrence (≤24 months post-surgery); and group C, patients who developed late recurrence (50–60 months post-surgery). Table 1 summarizes the clinical and pathological data of the study population. Except for group C, the cohort was balanced for the 4 intrinsic subtypes luminal A, B, basal-like and HER2+. The two later subtypes are associated with a more aggressive phenotype and higher risk of relapse [24], [26] which explains why group C (late recurrence) was mainly composed of luminal tumors (Table 1).

**Table 1:** Clinical and pathological features of the study population.

		Group A*		Group B*		Group C*		p-val
		n	(%)	n	(%)	n	(%)	
**Number of patients**		36	(48.0)	27	(36.0)	12	(16.0)	
**Age**	≤50	15	(45.5)	10	(37.0)	4	(36.4)	0. 8034
	>50	18	(54.5)	17	(63.0)	7	(63.6)	
**Hormonal status**	Preperim.**	15	(41.7)	10	(37.0)	6	(50.0)	
	Postmen.**	21	(58.3)	15	(55.6)	6	(50.0)	
	Unknown	0	(0.0)	2	(7.4)	0	(0.0)	
**Tumor size (cm)**	<2	5	(15.2)	2	(7.4)	3	(27.3)	0. 7487
	2–5	22	(66.7)	17	(63.0)	7	(63.6)	
	>5	5	(15.2)	4	(14.8)	1	(9.1)	
	Unknown	1	(3.0)	4	(14.8)	0	(0.0)	
**Tumor stage**	I	3	(8.3)	4	(14.8)	1	(8.3)	
	II	19	(52.8)	8	(29.6)	8	(66.7)	
	III	14	(38.9)	15	(55.6)	3	(25.0)	
**Hystological grade**	1	4	(11.1)	0	(0.0)	0	(0.0)	
	2	14	(38.9)	16	(59.3)	9	(75.0)	
	3	16	(44.4)	8	(29.6)	2	(16.7)	
	Unknown	2	(5.6)	3	(11.1)	1	(8.3)	
**Histologic subtype**	Lobulillar	4	(11.1)	0	(0.0)	0	(0.0)	
	Ductal	29	(80.6)	24	(88.9)	11	(91.7)	
	Medullar	0	(0.0)	1	(3.7)	0	(0.0)	
	Carcinoma	1	(2.8)	2	(7.4)	0	(0.0)	
	Mixed	2	(5.6)	0	(0.0)	1	(8.3)	
**Intrinsic subtype**	Luminal A	9	(25.0)	3	(11.1)	7	(58.3)	
	Luminal B	9	(25.0)	6	(22.2)	3	(25.0)	
	Basal-like	9	(25.0)	9	(33.3)	1	(8.3)	
	HER2-enriched	9	(25.0)	9	(33.3)	1	(8.3)	
**Type of surgery**	Conservative	22	(61.1)	9	(33.3)	5	(41.7)	
	Radical	14	(38.9)	18	(66.7)	7	(58.3)	
**Affected lymph node**	Negative	14	(42.4)	18	(48.6)	5	(45.5)	0.0292
	1–3	13	(39.4)	5	(13.5)	5	(45.5)	
	≥4	6	(12.8)	14	(37.8)	1	(9.1)	
**Therapy**	Chem.***	28	(77.8)	23	(85.2)	7	(58.3)	
	Horm.***	20	(55.6)	15	(55.6)	10	(83.3)	
	Rad.***	25	(69.4)	13	(48.1)	6	(50.0)	

*Group A  =  no recurrence, Group B  =  early recurrence (≤24 months after surgery), Group C  =  late recurrence (50–60 months after surgery).

**Preperim.  =  Pre-perimenopausic, Postmen.  =  postmenopausic.

***Chem.  =  chemotherapy, Horm.  =  hormonotherapy, Rad.  =  radiotherapy.

**Figure 1:**
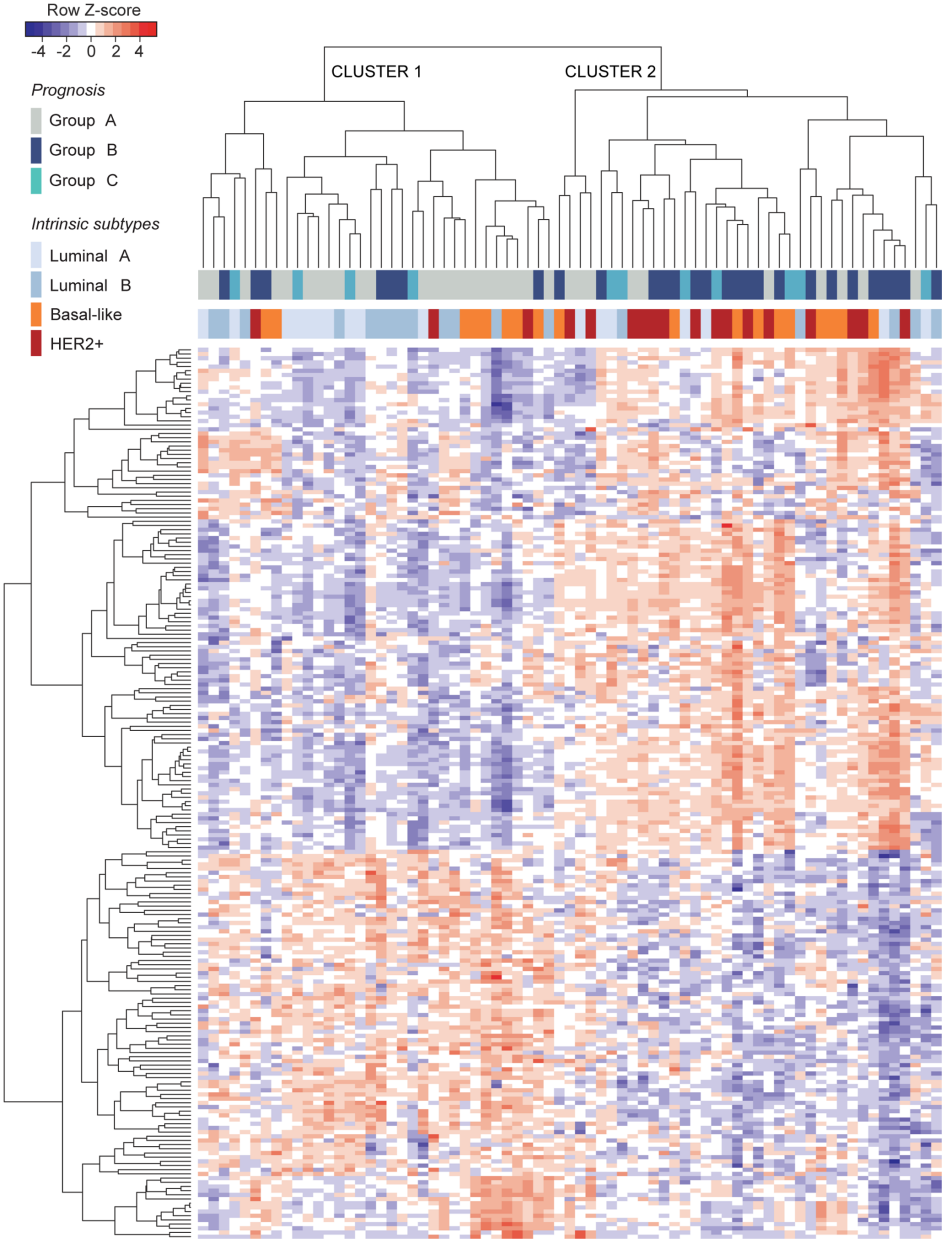
MicroRNA expression profiles in primary breast tumors from patients with different prognosis. Total RNA was obtained from 71 breast tumors, converted to cDNA and hybridized to Affymetrix miRNA Chip Array 2.0. After normalization, differential miRNA expression data was analysed by unsupervised hierarchical clustering. Color bars on top of the heatmap refer to the prognostic group and intrinsic subtype of each tumor. Group A included tumors from patients who were disease-free ≥60 months after surgery, group B included tumors from early-relapsing patients (≤24 months) and group C included tumors from late-relapsin patients (50–60 months after surgery). Tumors grouped in two main clusters (cluster 1 and cluster 2), showing opposite expression profiles and strongly associated with prognosis groups. Thus, cluster 1 included most luminal and/or non-relapsing tumors while cluster 2 mostly included basal-like and/or early-relapsing tumors.

Microarrays were first analyzed for quality control and normalized for miRNA expression (see methods). Four samples with a poor signal quality were excluded from the study, leaving a cohort of 71 breast tumors for further analysis. Unsupervised hierarchical clustering of the microarray data showed that the transcription profiles of miRNAs discriminate the prognosis groups in two distinct clusters (Figure 1). Cluster 1 included 70% of all tumors from group A and 26% of all tumors from group B while cluster 2 included 74% of all tumors from group B and 30% of all tumors from group A. Overall, tumors from group C were distributed within clusters 1 and 2, with a slightly greater proportion (63%) grouped in cluster 2. Since group C represents a clinical outcome intermediate between no recurrence (group A) and early recurrence (group B), the wide distribution of tumors from group C within clusters 1 and 2 could reflect that variation at the molecular level. An alternative explanation is that group C cannot not be identified by a distinct miRNA expression profile due to either its intrinsic molecular nature or the lower sample size. Of note, tumors tend to cluster according to their ER status and thus, most luminal tumors (ER+) were grouped in cluster 1 while cluster 2 mainly included HER2+ and basal-like tumors, which are both ER- (Figure 1). Multiple pairwise comparison tests showed that the largest expression differences occurred between luminal A and basal-like tumors. Consequently, the largest list of candidate miRNAs was obtained after comparing luminal A versus basal-like or basal-like versus the other subtypes (Supplementary Table S1). Overall, these results suggest that the three groups of tumors (A, B and C) represent distinct biological entities. They are also in accordance with accumulating evidence indicating that miRNA signatures can be associated to intrinsic molecular subtypes, supporting its use as a valuable tool for cancer diagnosis and prognosis [3], [43], [70], [71].

In order to select the statistically significant and differentially expressed miRNAs from Fig. 1, paired and multiple comparisons among the prognosis groups A, B and C were performed. Two different approaches, limma and RankProd Bioconductor, were employed. Only those candidates with a fold change (FC)>2 (either up- or down-regulated) and an adjusted p-value<0.05 were selected (Table 2). Thus, comparison of the logFC and p-values obtained with both limma and RankProd libraries led to the identification of miR-149, miR-20b, miR-30a-3p, miR-342-5p, miR-625 and miR-10a as the miRNAs that most significantly changed their expression when comparing tumors from disease-free patients versus relapsing patients, i.e. group B vs A or BC vs A (Table 2). As we had observed in the hierarchical clustering (Figure 1), the largest differences in expression of the six miRNAs were again detected when comparing B vs A (Table 2). In contrast, paired comparisons of either group A or B with the group C did not result in any statistically significant miRNA. Notably, the relative levels of all the candidate miRNAs were lower in samples from group B compared to the others, suggesting that these miRNAs could act, directly or indirectly, as suppressors of metastasis. Other researchers have also observed a general down-regulation of miRNA levels in breast cancer [72].

**Table 2:** Most significant deregulated miRNAs in breast tumors from relapsing patients.

		limma F*		RankProd**		RT-qPCR***	
Comparison#	miRNA	logFC	adj-pval	logFC	adj-pval	logFC	SE
**B/A**	hsa-miR-149	−1.410	0.0016	−1.615	<0.00001	−2.646	0.724
	hsa-miR-20b	−1.048	0.0071	−1.237	<0.00001	−1.542	0.521
	hsa-miR-30a-3p	−1.359	0.0078	−1.521	<0.00001	−1.001	0.514
	hsa-miR-625	−1.149	0.0014	−1.377	<0.00001	−0.347	0.282
	hsa-miR-10a	−1.235	0.0168	−1.547	<0.00001	−1.108	0.404
**BC/A**	hsa-miR-149	−1.120	0.0117	−1.329	<0.00001	−2.555	0.681
	hsa-miR-20b	−1.016	0.0076	−1.155	<0.00001	−1.470	0.536
	hsa-miR-30a-3p	−1.124	0.0256	−1.326	<0.00001	−0.994	0.458
	hsa-miR-625	−1.003	0.0049	−1.223	<0.00001	−0.266	0.237
**B/AC**	hsa-miR-149	−1.294	0.0052	−1.446	<0.00001	−2.340	0.698
	hsa-miR-10a	−1.397	0.0093	−1.647	<0.00001	−1.241	0.404
	hsa-miR-342-5p	−1.123	0.0159	−1.254	<0.00001	−1.194	0.627

# Group A  =  no recurrence, Group B  =  early recurrence (≤24 months after surgery), Group C  =  late recurrence (50–60 months after surgery).

*limma F, analysis of filtered data (sd>70%) using limma.

**RankProd, analysis of unfiltered data using RankProduct algorithm.

***RT-qPCR, Relative miRNA expression was calculated using the ΔΔC_t_ method. The standard error (SE) was calculated based on the theory of error propagation [107].

Regarding the intrinsinc subtypes, we found lower levels of miR-149, miR-30a-3p and miR-342-5p in ER- tumors (Supplementary Table S1). In that respect, others have shown repression of miR-149 levels in basal-like and HER2+ tumors [70], [73], [74]. and overexpression of miR-342-5p in ER+ breast tumors [75]. Jansen et al. found an association between miR-342-5p and ER expression in lymph node negative breast disease, with a strong downregulation in basal-like tumors. They also showed an inverse relationship between the mitotic index and both miR-30a-3p and miR-342-5p [76].

**Figure 2:**
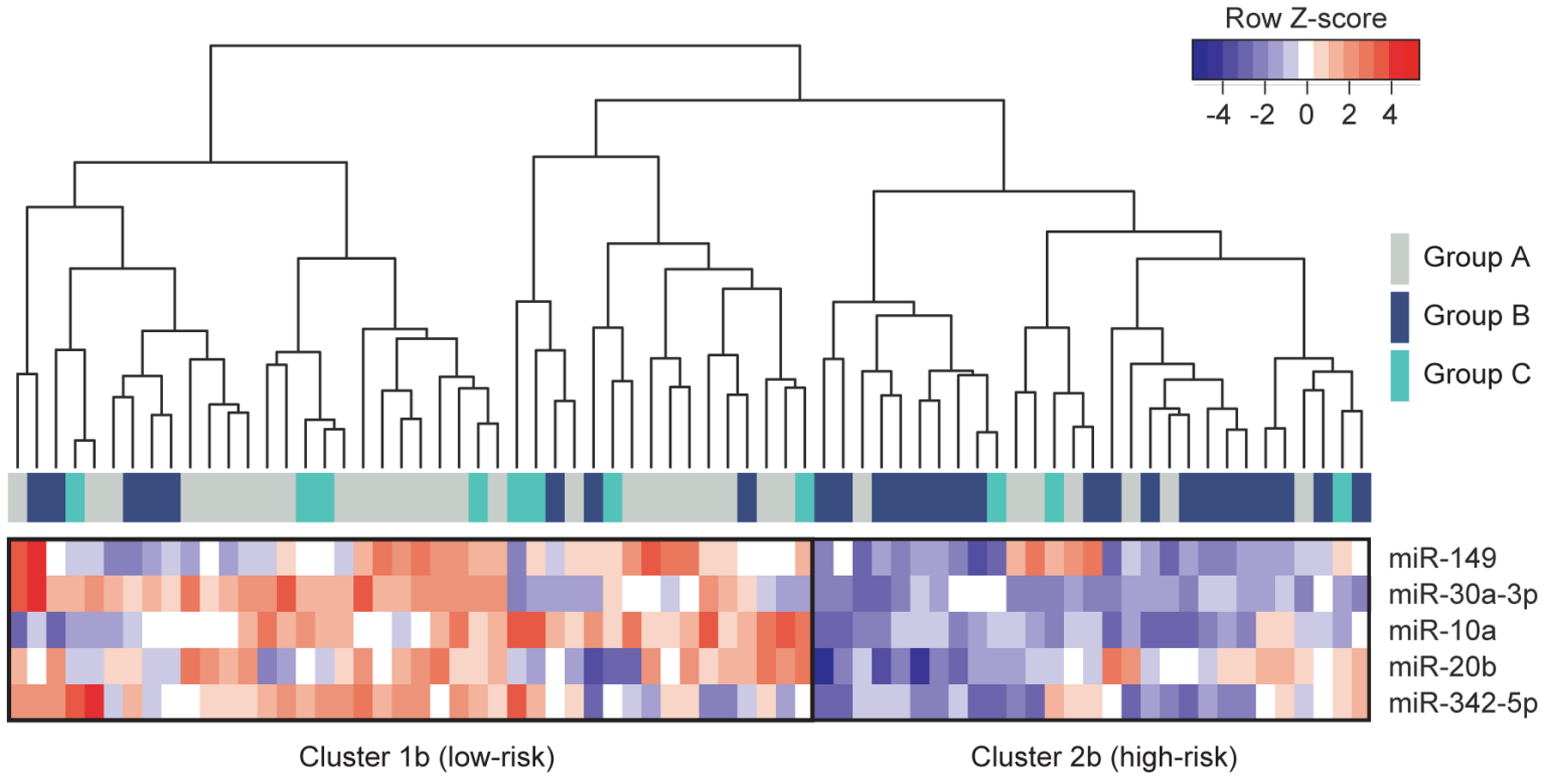
A 5-miRNA signature is associated with early recurrence in breast cancer. Hierarchical clustering of the 71 tumor samples based on expression of the 5-miRNA signature. Note that lower expression levels of the 5-miRNA signature defines a distinct cluster 2b wich mainly includes tumors from “high risk” patients (group B). On the contrary, most patients with good prognosis (group A) had tumors with normal or higher-than normal levels of the 5-miRNA signature, defining a different cluster 1b (“low risk”).

Differential expression of all six miRNAs were also determined by RT-qPCR in the three prognosis groups (Table 2). With the exception of miR-625, which could not be validated, miR-149, miR-20b, miR10a, miR-30a-3p and miR-342-5p (the “5-miRNA signature”, from now on) were all confirmed to be down-regulated in tumors from relapsing patients (groups B or C) when compared with tumors from relapse-free patients (group A, Table 2). MiR-625 was excluded from any further studies since RT-qPCR data showed minimal variation between groups (FC<2). Next, we re-clustered the 71 tumors based on the 5-miRNA signature. As shown in Figure 2, tumors from groups A and B were clearly segregated in two distinct clusters, which included most of the expected samples in each category: 78.8% group A in cluster 1b (low risk) and 70.4% group B in cluster 2b (high risk). Of note, the supervised analysis included most tumors from group C (72.8%), in cluster 1b, indicating that the 5-miRNA signature specifically discriminates tumors with an overall higher risk of early recurrence.

### The 5-miRNA signature

MiR-149 was the most significant miRNA downregulated in group B, as determined by microarray hybridization and by RT-qPCR. This miRNA has been described as a TS-miR that regulates the expression of genes associated with cell cycle, invasion or migration and its downregulation has been observed in several tumor diseases, including gastric cancer and breast cancer [70], [77]–[81]. Down-regulation of miR-149 can occur epigenetically, by hypermethylation of the neighbouring CpG island [80] or by impaired processing of the pri-miR-149 precursor, in a polymorphic variant [79]. In a recent work, downregulation of miR-149 has been associated with elevated levels of the transcription factor SP1, increase invasiveness and lower 5-year survival in colorectal cancer [80]. The p53 repressor ZBTB2 is also a target of miR-149 [81], which could explain, at least partially, its function as a TS-miR.

MiR-30a-3p is a member of the miR-30 family, which is associated with mesenchymal and stemness features [82], [83] and is downregulated in several types of cancer [84]–[86]. Recently, Rodriguez-Gonzalez et al. have linked low levels of this miRNA to tamoxifen resistance in ER+ breast tumors. They have also proposed several targets of miR-30a-3p involved in proliferation and apoptosis, such as BCL2, NFκB, MAP2K4, PDGFA, CDK5R1 and CHN1 [87].

Regarding miR-20b, this miRNA is part of the miR-106b-363 cluster, which is frequently deregulated in cancer [88]–[91]. The levels of miR-20b associate with histological grade in breast cancer [92], [93]. This miRNA has been involved in regulating several key proteins such as ESR1, HIF-1α, VEGF or STAT3 [92], [94], [95]. In particular, because it targets both HIF-1α and VEGF and HIF-1α negatively controls miR-20b levels, it has been defined as an anti-angiogenic miRNA [95].

Both oncogenic and tumor suppressor features have been reported for miR-10a [96]. Thus, reduced expression of miR-10a has been associated with MAP3K7- and βTRC-mediated activation of the proinflammatory NFκB pathway [97]. Also, miR-10a downregulation represses differentiation in part by deregulation of the histone deacetylase HDAC4 [98] and positively affects invasiveness by de-repressing several members of the homeobox family of transcription factors [99].

Regarding miR-342-5p, it appears significantly deregulated only when we compare B vs AC (Table 2). Together with its counterpart (miR-342-3p), it is deregulated in inflammatory breast cancer [74] and its low expression has been associated with lower post-recurrence survival [100], likely because it targets AKT1 mRNA [101].

In sum, the available bibliographic data suggests that down-regulation of miR-149, miR-30a-3p, miR-20b, miR-10a and miR342-5p in primary breast tumors could confer them enhanced proliferative, angiogenic and invasive potentials.

#### Prognostic value of the 5-miRNA signature

**Figure 3:**
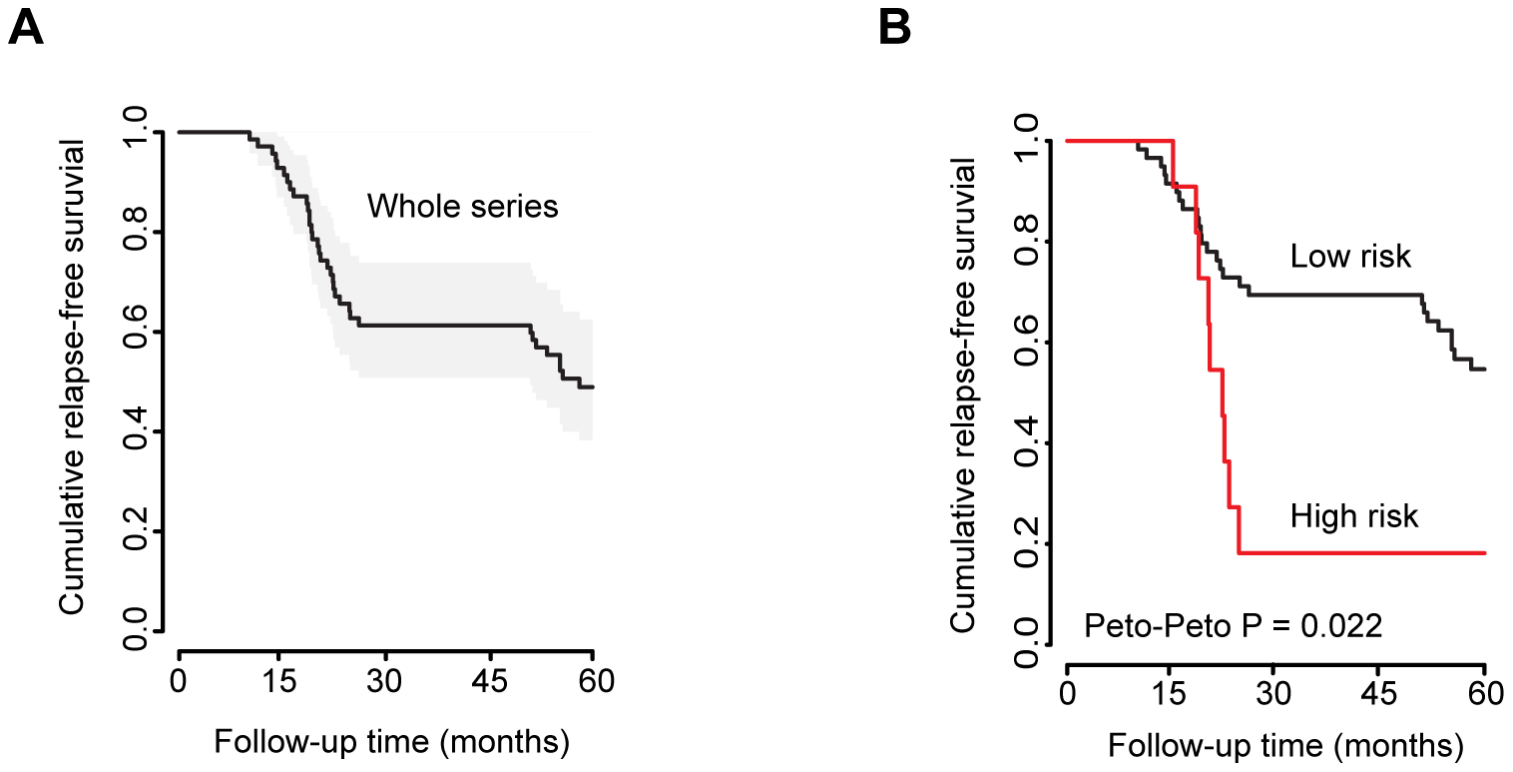
The 5-miRNA signature discriminates patients with diferent RFS. **A**) Kaplan-Meier graph for the whole patient cohort included in this study. **B**) Those patients whose tumors showed an overall down-regulation of the 5-miRNA signature (i.e. those from cluster 2b in Fig. 2) were classified as “high risk” (red line) and their cumulative RFS was calculated (red line). RFS was also calculated for the remaining patients in the cohort (“low risk”, black line). The Kaplan-Meier plot shows that the 5-miRNA signature specifically discriminates tumors with an overall higher risk of early recurrence.

The relationship between expression of the 5-miRNA signature and RFS was examined by a survival analysis. Figure 3A shows a Kaplan-Meier graph for the whole series of patients included in the study. Due to the intrinsic characteristics of the cohort, decreases in the RFS are only observed in the intervals 0–24 and 50–60 months (corresponding to groups B and C, respectively). We next grouped the tumors according to their 5-miRNA signature status in two different groups. One group included those tumors with all five miRNAs simultaneously downregulated, (FC>2 and p<0.05) and a second group included those tumors not having all five miRNAs downregulated. A survival analysis was performed using clinical data from the corresponding patients. As shown in Figure 3B, the Kaplan-Meier graphs for the two groups demonstrate that the 5-miRNA signature defines a “high risk” group of patients with a shorter RFS (Peto-Peto test with p-value = 0.02, when comparing the low vs high risk groups).

**Figure 4:**
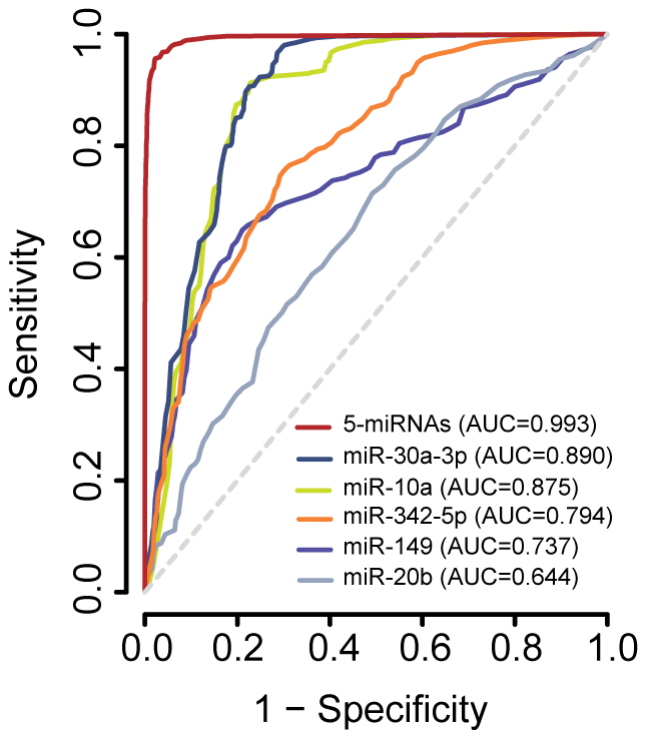
Receiver operating characteristic curve (ROC) for early breast cancer recurrence by the 5-miRNA signature status. ROC curves generated using the prognosis information and expression levels of the 5-miRNA signature can discriminate between patients who will develop early recurrence and those who will remain free of disease. Note that, although miR-30-3p and miR10a, individually have a high area under the curve (AUC) score, the 5-miRNA signature has the strongest predictive value (AUC = 0.993) to discriminate those patients likely to recur early (group B in our cohort).

Using a Cox proportional hazard regression model, we also tested all possible combinations of different covariates (tumor subtype, patient age, tumor size, number of lymph nodes affected and the 5-miRNA signature) with early relapse (≤24 months) to identify the best prognostic factors. The best model according to the AIC criterion included the tumor size and expression of the 5-miRNA signature (data not shown). Only the 5-miRNA signature (all five miRNAs down-regulated) resulted statistically significant in the Cox model for the high risk group (p-value = 0.02 with HR = 2.73, 95% CI: 1.17–6.36). The 5-miRNA expression data were also used to develop a predictor model through bootstrapping over a Naive Bayes classifier (B = 200 with N = 71, see methods). The prognostic accuracy of the models was assessed by a receiver operating charateristic (ROC) test (Figure 4). Considered individually, miR-30a-3p and miR-10a showed a strikingly high Area Under the Curve (AUC) score (0.890 and 0.875, respectively). This result suggests that mRNA targets regulated by miR-30a-3p and miR-10a could potentially add a greater contribution to the final outcome of the disease. However, the 5-miRNA signature had the strongest predictive value to discriminate tumors from patients that will develop early relapse (group B) from those that will remain free of disease (group A), with an AUC = 0.993 (Figure 4). In summary, the 5-miRNA signature has a good performance as a risk predictor for early breast cancer recurrence.

#### Candidate targets for the 5-miRNA signature

**Figure 5:**
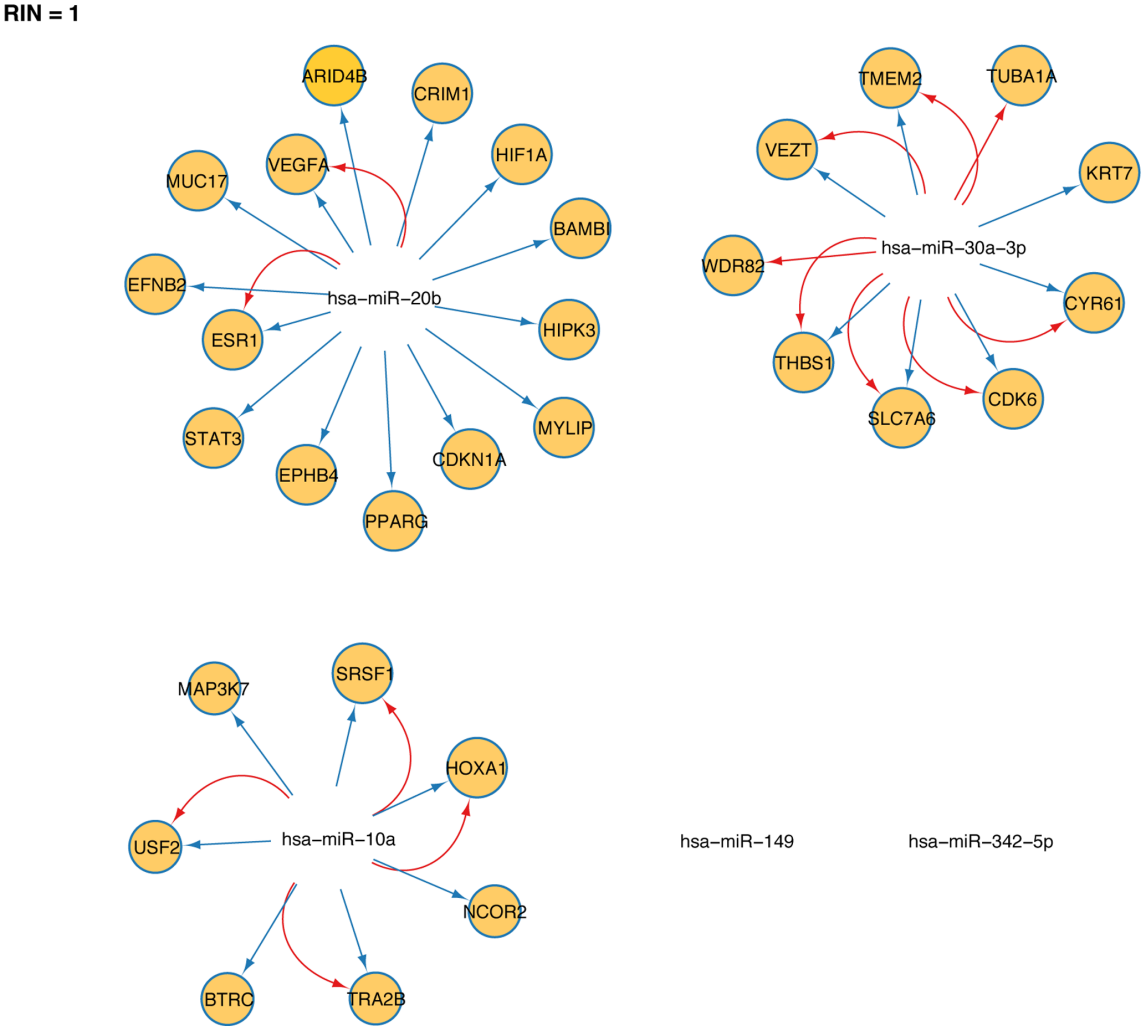
Prediction of mRNA targets likely to be regulated by the 5-miRNA signature. Biological networks were created using the Cytoscape software. Each network includes two types of nodes: the five individual miRNAs included in the 5-miRNA signature and their predicted mRNA targets (yellow circles), obtained from two different public databases (miRTarBase and miRecords). The number of databases included in the analysis defines the regulatory interaction network (RIN) threshold. Thus, at RIN = 1 the network includes all mRNA targets that appear in, at least, one database. The databases included in the RIN are identified by the color of the connecting arrows: miRTarBase (blue) and miRecords (red). Although many mRNAs are potential targets for miR-149 and miR-342-5p, the miRTarBase and miRecords versions included in this study did not reveal any targets experimentally validated for the two miRNAs.

To extend our set of five miRNAs with regulatory information, we next took advantage of the existing public databases curating predicted and validated miRNA-target interactions (MTIs). In particular, validated targets were obtained from the miTarBase and miRecords repositories (see methods). First, we created a biological network in Cytoscape [66] containing all the individual miRNAs included in the 5-miRNA signature (miR-149, miR-20b, miR10a, miR-30a-3p and miR-342-5p). Next, we extended the network by adding *H. sapiens* MTI data retrieved from the indicated repositories and, finally, extended regulatory interaction networks (RIN) were generated and visualized in Cytoscape. Each regulatory interaction in the network consist of two nodes, a regulatory component (miRNA) and a target biomolecule (mRNA) connected through one directed edge. Figure 5 shows the extended network when the RIN threshold was set to 1 (i.e. each predicted target appears in, at least, one RIN). Thus, at RIN = 1 the network included 14 validated targets assigned to miR-20b (VEGFA, BAMBI, EFNB2, MYLIP, CRIM1, ARID4B, HIF1A, HIPK3, CDKN1A, PPARG, STAT3, MUC17, EPHB4, and ESR1), 7 validated targets assigned to miR-10a (HOXA1, NCOR2, SRSF1, SRSF10/TRA2B, MAP3K7, USF2 and BTRC) and 9 validated targets assigned to miR-3a-3p (THBS1, VEZT, TUBA1A, CDK6, WDR82, TMEM2, KRT7, CYR61 and SLC7A6) (Figure 5). Taking these results into account and considering that i) the extended network was constructed with the 5-miRNA signature as the network nodes and ii) all MTIs depicted in Figure 5 have been experimentally verified, we suggest that at least some of the 30 mRNAs (Figure 5) could be regulated *in vivo* by the 5-miRNA signature in early-relapsing tumors.

**Figure 6:**
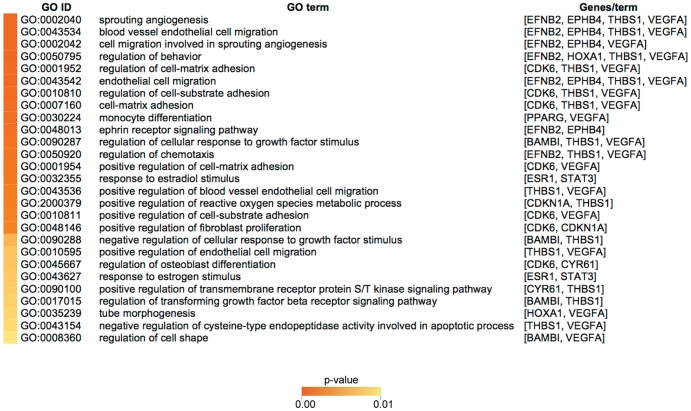
Gene Ontology (GO) terms associated with the predicted mRNA targets of the 5-miRNA signature. A GO term analysis was performed using terms of the “biological process” vocabulary. Shown are the GO identification number (GO ID), the name of the biological process (GO term) and the mRNA targets associated to each particular GO term. Only term ontologies with experimental evidence and corrected p-value≤0.01 are shown.

To gain further insight into the molecular basis of the 5-miRNA signature prognostic value, we investigated the biological pathways associated with the 30 experimentally verified targets from Figure 5. To that end, we searched for Gene Ontology (GO) terms and Kyoto Encyclopedia of Genes and Genomes (KEGG) pathways associated with the 30 targets as a whole set. It should be noted, however, that our restrictive approach –including only experimentally validated miRNA targets-, left miR-149 and miR-342-5p out of the GO analysis and therefore, additional biological pathways could be affected by downregulation of the 5-miRNA signature. To increase the predictive value of the GO analysis we considered only term ontologies with experimental evidence and p-value≤0.01. Interestingly, most targets in the set were associated with GO terms related to angiogenesis and cell migration (GO:0001954, GO:0002040, GO:0002042, GO:0043534 and GO:0043536), in addition to the GO terms “response to stradiol stimulus” (GO:0032355), “monocyte differentiation” (GO:0030224) and “ephrin receptor signaling pathway” (GO:0048013) (Figure 6). Other GO terms of particular relevance to our study were: “positive regulation of fibroblast proliferation” (GO:0048146), “regulation of chemotaxis” (GO:0050920), “regulation of cellular response to growth factor stimulation” (GO:0090287) and “positive regulation of reactive oxygen species metabolic process” (GO:2000379). Taken together, the computational analysis of putative, experimentaly verified mRNA targets for the 5-miRNA signature and their associated GO terms (p-value≤0.01) suggest that early recurrence in breast cancer is a consecuence of the higher angiogenic, invasive, and proliferative potential of a subset of tumors with downregulated levels of, at least, miR-20b, miR-10a and miR-30a-3p (Figure 5). In fact, integration of the GO terms into the KEGG pathway maps, provides further support for this notion, as the net effect of changes in the regulatory pathways affected by a rise in the predicted targets would be an increase in both proliferation and angiogenesis (Supplementary Figure S1).

**Figure 7:**
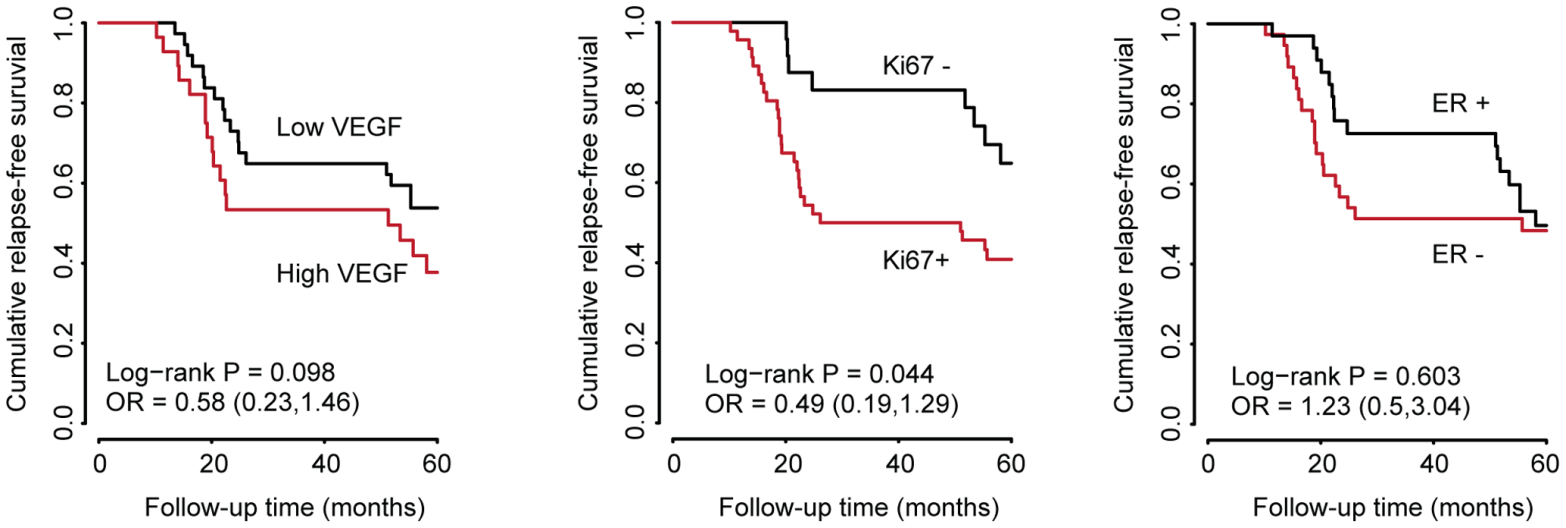
Patients with a higher risk of relapse have tumors with increased proliferative capacitity. The angiogenic (VEGF), proliferative (Ki67) and hormone receptor (ER) status of the primary breast tumors were assessed by immunohistochemistry with specific antibodies. Interpretation of the immunohistochemical signal (low/high for VEGF and positive/negative for Ki67 and ER) followed the criteria specified in the methods section. Patients were classified according to the VEGF, Ki67 and ER status of their tumors and the cumulative RFS was calculated. The Kaplan-Meier plots show a reduced RFS in patients with tumors highly positive por VEGF, positive for Ki67 and negative for ER, although the differences were only statistically significant for Ki67 (Log-rank P = 0.044). All 71 tumors included in this study were processed for Ki67 and ER staining while only 67 could be processed for VEGF staining.

In an effort to validate such hypothesis, we collected retrospective immunohistochemical data for expression of angiogenesis (VEGF) and proliferation (Ki67) markers in the set of primary tumors (Table 1), when available. As a positive control, we also included estrogen receptor (ER) expression data, as it is often associated with prognosis [3], [4]. Since down-regulation of a miRNA should result in an increased stability of its target mRNAs, we anticipated an increased expression of VEGF and Ki67 in those tumors identified by the 5-miRNA signature (high risk group). Quantification of VEGF, Ki67 and ER immunostaining was performed as previously described [23], [50], [51] and the percentage of tumors showing low or high expression of each marker was calculated for each prognostic group (A, B or C) or the 5-miRNA signature status (low or high risk). The results of the analysis are summarized in Table 3. We only found a statistically significant association when comparing Ki67 vs prognostic groups (p-value = 0.012), ER vs prognostic groups (p-value = 0.025) or ER vs risk groups (p-value<0.0001). In contrast, VEGF expression was not found to be significantly associated with either the prognostic groups or the 5-miRNA signature (Table 3). In spite of that, we found slightly increased levels of VEGF and Ki67 in early-relapsing tumors (group B) and in the “high risk” group (Table 3). A survival analysis also showed a reduced RFS in those patiens with tumors positive for Ki67, negative for ER and with increased expression of VEGF (Figure 7). Again, however, only Ki67 levels were signficantly associated with RFS (P = 0.044, Figure 7, middle panel). We suggest that the lack of statistically significant association between VEGF levels and the 5-miRNA signature or RFS could be a consequence of the relatively small number of samples included in our immunohistochemical analysis. Future studies with a larger number of tumors will address the contribution of VEGF expression levels to early relapse. Regardless of that, our data demonstrate that tumors with the worst prognosis (group B) had a statistically significant higher proliferative potential, as measured by Ki67 immunostaining.

**Table 3:** Expression levels of VEGF, Ki67 and ER in tumors.

		Group				Risk Level		
		A	B	C		Low	High	
		n (%)	n (%)	n (%)	p-val	n (%)	n (%)	p-val
**VEGF**	Low	6 (20.7)	4 (15.4)	1 (9.1)	NS	14 (37.8)	5 (17.2)	NS
	High	23 (79.3)	22 (84.6)	10 (90.9)		23 (62.2)	24 (82.8)	
**Ki67**	Negative	15 (45.4)	4 (14.8)	6 (54.5)	0.012	17 (40.5)	8 (25.6)	NS
	Positive	18 (54.6)	23 (85.2)	5 (45.5)		25 (59.5)	21 (72.4)	
**ER**	Negative	17 (51.5)	18 (66.7)	2 (18.2)	0.025	14 (33.3)	23 (79.3)	<0.0001
	Positive	16 (48.5)	9 (33.3)	9 (81.8)		28 (66.7)	6 (20.7)	
								

NS: Not significant (p-value was calculated using a Fisher's exact test).

### Biological significance

Predicting early relapse represents a major challenge in the clinical practice, because an early failure very often corresponds to a more aggressive disease with fewer therapeutic options and poorer outcome. Indeed, up to half of all the relapses reside within the early peak of recurrence described by Demicheli and colleagues [16]. According to their model of bimodal hazard function, early recurrence can be explained as an iatrogenic effect of surgically removing the primary tumor. Late recurrences, on the contrary, are not synchronized and therefore not influenced by the surgical procedure. Instead, they are proposed to result from the sudden growth of single-cell micrometastases during the natural evolution of the disease [11]. Following this hypothesis, previous reports have focused on dormant metastatic foci, their surrounding microenvironment or even serum signals, to find factors that could explain different risks of recurrence. In this regard, different groups -including ours- have observed an association between recurrence and post-surgery variations in several circulating inflammatory cytokines [17]–[19].

The model of bimodal recurrence relies on the concept of tumor dormancy and metastatic homeostasis being perturbed by tumor removal [11], [12]. Tumor cells leave the primary site to seed in a different, distant tissue where they remain dormant for a variable period of time, either as single cells or as micrometastasis [102]–[104]. Most micrometastases do not proliferate actively and only a small number (<10%) have an angiogenic phenotype [11]. Non-angiogenic micrometastases remain quiescent in the absence of an angiogenic switch and even angiogenic micrometastases cannot grow to more than avascular foci without proper neovascularization. Dormancy then results from a balance between pro- and anti-angiogenic signals that affect the micrometastases. Genetic alterations acquired over the natural course of the disease will eventually produce an imbalance between pro- and anti-angiogenic factors favoring neovascularization and growth of the micrometastatic foci (the angiogenic switch) [103]. The model of bimodal recurrence assumes that the primary tumor contributes to the homeostasis of distant metastases by releasing anti-angiogenic factors that keep angiogenic metastatic cells in a dormant, avascular state. Surgical removal of the tumor would abolish the angiogenic restrain and favor the metastatic process. In addition, growth factors and cytokines released as a consequence of tissue wounding during the surgery would add and angiogenic spike, boosting the metastatic process [9], [11], [13]. Here, we have explored the hypothesis that, in addition to such an iatrogenic effect, tumors likely to relapse early after surgery have distinct, intrinsic molecular characteristics that favor metastatic growth.

Several miRNAs with prognostic value for metastatic breast cancer have been proposed [44], [93], [105], [106]. In all cases, however, recurrence was considered as a homogeneous process. Unlike most authors, we have considered recurrence as proposed by Demicheli and coworkers: a bimodal, heterogeneous process influenced by both the natural history of the disease and the surgical removal of the primary tumor [13]. We have shown here that primary tumors from early-relapsing patients are dissimilar to tumors from disease-free patients, at least concerning to their miRNA profile. In our opinion, these differences in miRNA expression –which should also impact on the tumor transcriptome– reflect two distinct biological entities with, at least, different proliferative potential (Figure 7). Our network analysis predicted several targets that could also confer enhanced angiogenic and invasive capacity to the early-relapsing tumors. However, we could only prove a statistically significant correlation between early recurrence and proliferation, as measured by Ki67 statining.

Our study neither support nor refute an iatrogenic effect derived from surgery but rather demonstrates that breast tumors with different risk or relapse can be differentiated at the miRNA expression level. However, if we consider such an iatrogenic effect, our data could be reconciled with a bimodal recurrence distribution as follows: the increased proliferation potential of the tumors in group B (early recurrence) could lead to the formation of micrometastasis as opposed to the tumors with lower proliferative capacity (group A and some in group C), which would seed mainly as single-cell micrometastasis. Both metastatic foci would remain avascular and therefore dormant, until a surgery-driven angiogenic switch restarts tumor progression. Then, micrometastasis would grow at different rates based on their distinct proliferative potential (including some not growing at all). Differences in the angiogenic and invasive capacities of the tumors could also contribute to the temporal distribution of recurrence but they still need to be firmly stablished in future studies.

### Conclusions

MiRNA deregulation is involved in breast cancer and there is a growing interest in the identification of miRNA signatures with biomarker potential in the clinical setting. Here, we have provided evidence that miRNA profiling can discriminate patients with different risk of relapse. In particular, we have identified a 5-miRNA signature (including miR-149, miR-30a-3p, miR-20b, miR-10a and miR-342-5p) with prognostic value (AUC = 0.993, p-value<0.05) that is downregulated in primary breast tumors from patients who develop early recurrence. In addition, we have identified a set of 30 mRNAs predicted to be up-regulated by the 5-miRNA signature in early-relapsing tumors. Notably, the set included mRNAs coding for proteins mainly involved in angiogenesis and proliferation (VEGFA, THBS1, EPHB4 CDK6 and DCKN1, among others). We have demonstrated that early-relapsing tumors have a significant higher proliferative potential, as measured by Ki67 immunostaining. Further efforts are needed to address the role of these biomarkers in the process of recurrence, but they could contribute to develop novel treatment strategies and to better understand the specific functions of miRNAs in cancer progression and metastasis.

Although our results may require further external validation in a larger cohort, we propose our set of 5 miRNAs as an independent prognostic-associated signature for early recurrence in breast cancer.

## Supporting Information

Figure S1 **Overview of predicted biological functions affected by expression of the 5-miRNA signature in early-metastasizing tumors.** The set of 30 predicted targets of the 5-miRNA signature (Fig. 5) was integrated into the Kyoto Encyclopedia of Genes and Genomes (KEGG) to generate a map of the key proteins (red stars) and pathways most likely associated with the targets. Note that an increase in the predicted targets (due to down-regulation of the 5-miRNA signature in early-relapsing tumors) would result in a net increase in proliferation and angiogenesis.
(EPS)

Table S1 **Most significant deregulated miRNAs in breast tumors of different intrinsic subtypes.** Only those miRNAs with a FC>2 and p<0.05 were included in the list.
(DOC)
