# Supplementary material for: A microRNA Signature Associated with Early Recurrence in Breast Cancer
Source: PLoS One. 2014 Mar 14;9(3):e91884. doi: 10.1371/journal.pone.0091884 (PMC3954835; doi:10.1371/journal.pone.0091884)
Supplement: Table S1 — Most significant deregulated miRNAs in breast tumors of different intrinsic subtypes. Only those miRNAs with a FC>2 and p<0.05 were included in the list. (DOC) [file pone.0091884.s002.doc]

Supplementary Table S1. Most significant deregulated miRNAs in breast tumors of different intrinsic subtypes

|  | **Luminal A vs Basal-like** | | **Lumina A vs HER2** | | **Luminal B vs Basal-like** | | **Luminal B vs HER2** | | **Luminal A vs others** | | **Basal-like vs others** | |
| --- | --- | --- | --- | --- | --- | --- | --- | --- | --- | --- | --- | --- |
| ID | logFC | adj.P.Val | logFC | adj.P.Val | logFC | adj.P.Val | logFC | adj.P.Val | logFC | adj.P.Val | logFC | adj.P.Val |
| hsa-miR-342-3p | 2,01 | 1,29E-07 | 1,89 | 8,31E-07 | 1,72 | 1,76E-05 | 1,60 | 0,00 | 1,42 | 1,02E-05 | -1,27 | 1,45E-04 |
| hsa-miR-342-5p | 2,22 | 2,52E-04 | 2,01 | 2,21E-03 | 1,95 | 2,38E-03 | 1,74 | 0,01 | 1,52 | 8,54E-03 | -1,45 | 4,47E-03 |
| hsa-miR-29b-2-5p | 1,45 | 7,39E-03 | 1,27 | 3,98E-02 | 1,92 | 4,73E-04 |  |  |  |  |  |  |
| hsa-miR-18a | -2,15 | 4,54E-04 |  |  | -1,61 | 2,81E-02 |  |  |  |  | 1,62 | 1,96E-03 |
| hsa-miR-934 | -1,51 | 1,00E-03 |  |  | -1,37 | 6,97E-03 |  |  |  |  | 1,37 | 4,19E-04 |
| hsa-miR-193b | 1,27 | 7,80E-03 |  |  | 1,36 | 7,97E-03 |  |  |  |  |  |  |
| hsa-miR-375 | 3,10 | 4,38E-03 |  |  | 3,60 | 1,33E-03 |  |  |  |  | -3,01 | 6,65E-04 |
| hsa-miR-146a | -1,60 | 2,26E-02 |  |  |  |  |  |  |  |  | 1,34 | 1,92E-02 |
| hsa-miR-362-5p | -1,38 | 4,61E-03 |  |  |  |  |  |  |  |  | 0,90 | 4,01E-02 |
| hsa-miR-20a | -1,27 | 1,55E-03 |  |  |  |  |  |  |  |  | 1,01 | 2,89E-03 |
| hsa-miR-17 | -1,32 | 2,52E-04 |  |  |  |  |  |  |  |  |  |  |
| hsa-miR-106a | -1,30 | 4,32E-04 |  |  |  |  |  |  |  |  |  |  |
| hsa-miR-505-5p | -1,29 | 4,38E-03 |  |  |  |  |  |  |  |  |  |  |
| hsa-miR-17-3p | -1,08 | 3,33E-02 |  |  |  |  |  |  |  |  |  |  |
| hsa-miR-30a |  |  | 1,86 | 4,91E-03 |  |  | 1,51 | 0,02 |  |  |  |  |
| hsa-miR-612 |  |  | -1,16 | 4,33E-02 |  |  |  |  |  |  |  |  |
| hsa-miR-1308 |  |  | -1,03 | 4,91E-03 |  |  |  |  |  |  |  |  |
| hsa-miR-30c |  |  | 1,14 | 4,12E-02 |  |  |  |  |  |  |  |  |
| hsa-miR-141 |  |  | 1,41 | 4,09E-02 |  |  |  |  |  |  |  |  |
| hsa-miR-30a-3p |  |  | 1,98 | 3,11E-02 |  |  |  |  |  |  |  |  |
| hsa-miR-224-3p |  |  |  |  |  |  | -1,29 | 0,03 |  |  |  |  |
| hsa-miR-125a-5p |  |  |  |  |  |  | 1,03 | 0,02 |  |  |  |  |
| hsa-miR-193b |  |  |  |  |  |  | 1,09 | 0,03 |  |  |  |  |
| hsa-miR-29b-2-5p |  |  |  |  |  |  | 1,74 | 0,00 |  |  | -1,17 | 9,67E-03 |
| hsa-miR-149 |  |  |  |  |  |  |  |  |  |  | -1,33 | 3,46E-02 |
| hsa-miR-425 |  |  |  |  |  |  |  |  |  |  | -0,77 | 4,01E-02 |
| hsa-miR-99b |  |  |  |  |  |  |  |  |  |  | -0,60 | 2,19E-02 |
| hsa-miR-1975 |  |  |  |  |  |  |  |  |  |  | -0,41 | 4,01E-02 |
| hsa-miR-18a-3p |  |  |  |  |  |  |  |  |  |  | 0,54 | 4,01E-02 |
| hsa-miR-18b |  |  |  |  |  |  |  |  |  |  | 0,73 | 1,92E-02 |
| hsa-miR-19b |  |  |  |  |  |  |  |  |  |  | 0,94 | 4,16E-02 |
